# Papilledema and Pseudopapilledema in Alagille Syndrome: A Case Report

**DOI:** 10.1080/01658107.2025.2495293

**Published:** 2025-05-21

**Authors:** Muhammad A Khan, Danica Joseph, Chamil Dayajeewa, Timothy Ang, Krishna Tumuluri, Kate Reid

**Affiliations:** a Translational Ocular Research and Immunology Consortium (TORIC), Westmead Institute of Medical Research, Sydney, Australia; b Department of Ophthalmology, Canberra Hospital, Canberra, Australia; c Medical Imaging Department, Royal Prince Alfred Hospital, Sydney, Australia; d Department of Ophthalmology, Westmead Public Hospital, Sydney, Australia; e John Curtin School of Medical Research, Australian National University, Canberra, Australia

**Keywords:** Alagille syndrome, papilledema, pseudopapilledema, posterior embyrotoxin, optic disc glial proliferation

## Abstract

Alagille syndrome (ALGS) is a rare, multisystem, autosomal dominant disorder of variable penetrance, typically dominated by the consequences of bile duct paucity and congenital heart disease. Neuro-ophthalmic findings include optic disc swelling and cerebral vascular anomalies. Here, we discuss the care of a lean young man with ALGS and extreme optic disc swelling. He had significant systemic co-morbidities, in the form of renal failure and anticoagulation after cardiac surgery. His disc swelling proved to be due to a combination of pseudopapilledema from ALGS glial proliferation with possible drusen, and true papilledema, with cerebrospinal fluid (CSF) opening pressure of 31 cm H2O at lumbar puncture.

Despite renally adjusted acetazolamide and topiramate, field loss beyond blind spot enlargement emerged. CSF shunting was deemed unwise, due to the high revision rate which so often follows. Bilateral optic nerve sheath fenestration was therefore undertaken, and succeeded in reversing the new field loss. Disc swelling did not decline dramatically, due to the ALGS pseudopapilledema as well as the presumed chronicity of the patient’s papilledema.

Since pseudopapilledema and papilledema can co-exist in ALGS, it is important to adequately distinguish them, ensuring that the emerging visual threat from true papilledema is not overlooked. Systemic comorbidities of the syndrome will need thoughtful care from a co-ordinated multidisciplinary team when treating the papilledema. Screening for cerebral aneurysm is another important principle in the care of patients with ALGS.

## Case presentation

**Figure 1.**
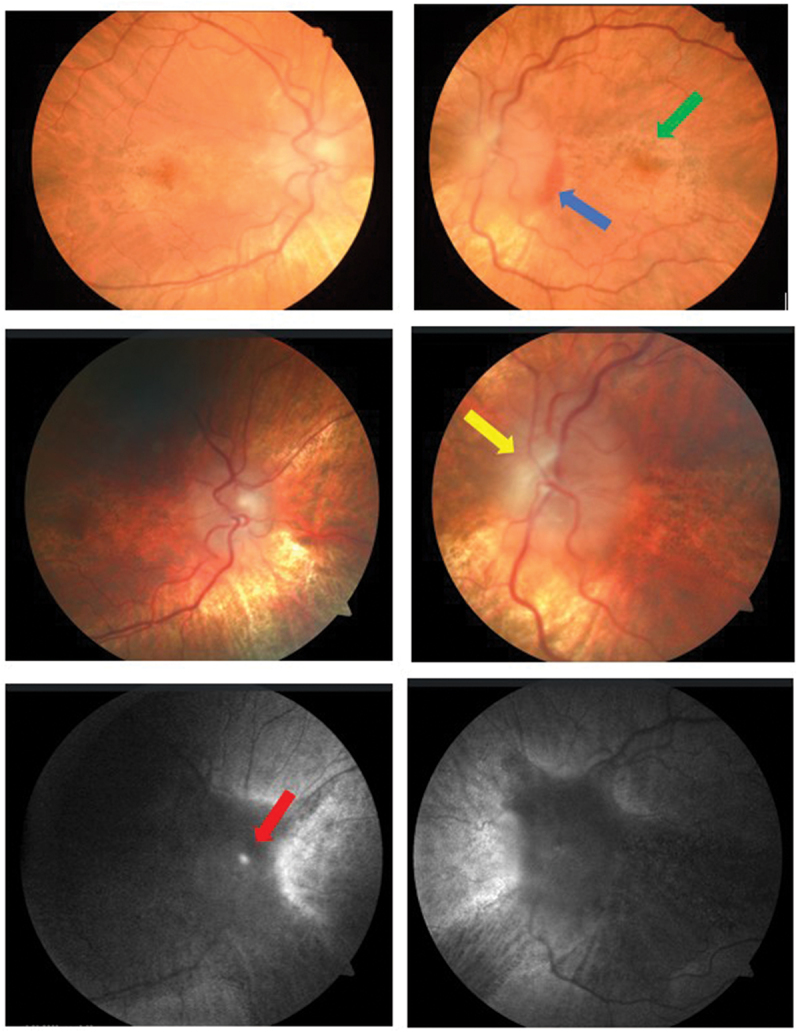
Optic discs at presentation. The gross bilateral disc swelling accounts for the blurred images.

**Figure 2.**
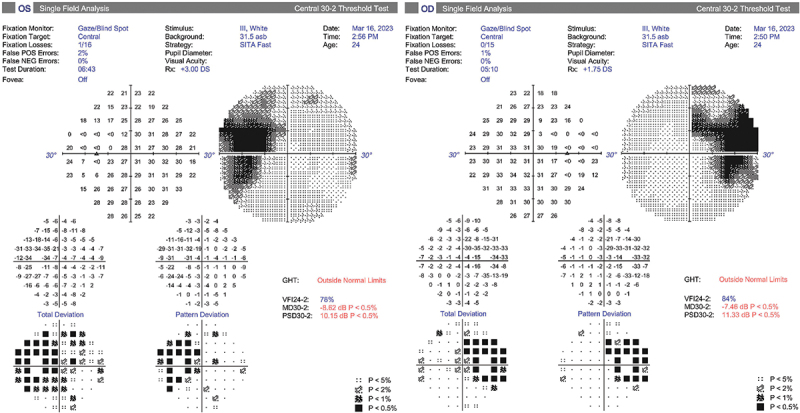
Visual fields at presentation. There was gross bilateral blind spot enlargement. The left peripapillary hemorrhage seen at presentation had resolved by the time these fields were obtained.

A 24-year-old Caucasian man with Alagille syndrome (ALGS) and a BMI of 16 kg/m^2^ presented to his optometrist with headache, profound optic disc swelling, and left peripapillary hemorrhage. Acuity was right 6/7.5 and left 6/9. There were no inflammatory signs on slit lamp examination. Optic nerve head drusen were absent on B-scan and CT; autofluorescence was thought to show curvilinear glial proliferation as well as possible drusen (Figure 1). Both the OCT enhanced depth imaging of the discs and measurements of the retinal nerve fiber layer thickness (RNFL) were unreliable due to the gross disc swelling, which markedly exceeded the highest Frisen grade. The maculae showed mottled hypopigmentation (Figure 1), and the macular ganglion cell layer (GCL) was reduced on OCT, with an average thickness of 50 microns bilaterally. Fluorescein angiography was not undertaken due to an eGFR of 26 mL/min/1.73 m^2^. The blind spots were grossly enlarged on the patient’s first ophthalmic visual field (Figure 2), but there was no other field loss, and at that time the peripapillary hemorrhage had resolved. As well as end-stage kidney disease (ESKD), the patient had a mechanical aortic valve replacement (AVR). Medications included warfarin, amlodipine, atorvastatin, sodium bicarbonate, allopurinol and calcitriol, and blood pressure was well-controlled. Iron levels were satisfactory.

Top row: There was left peripapillary hemorrhage (blue arrow) indicating an acute process. Fundus pigmentary mottling and hypopigmentation typical of ALGS were seen (green arrow). Middle row: Curvilinear glial proliferation was seen on the left (yellow arrow). This was uncalcified on B-Scan and CT and lacked autofluorescence. Bottom row: The autofluorescence seen on the right (red arrow) may have represented a drusen, but was uncalcified on B-Scan and CT.

**Figure 3.**
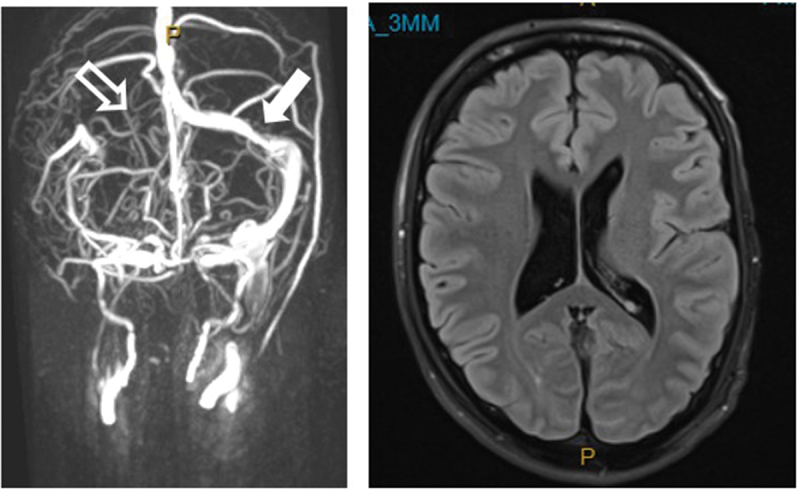
MR venogram brain at presentation. There was right transverse sinus aplasia (outlined arrow) and minor stenosis of the left transverse sinus (solid arrow). The ventricles were not compressed, as might be seen in IIH.

MR venogram brain showed aplasia of the right transverse sinus and minor stenosis of the left (Figure 3). It excluded aneurysm and optic nerve hyperintensity, but enhancement could not be assessed, as gadolinium was contra-indicated by the reduced eGFR. Catheter venography excluded cerebral venous sinus thrombosis and jugular vein abnormality, showing only mild venous hypertension (superior sagittal sinus 18 mmHg); the gradient across the left transverse sinus stenosis was normal (3 mmHg). Subsequent MR angiogram brain excluded cerebral aneurysm.

Warfarin was ceased via a heparin infusion, as enoxaparin was contra-indicated by the reduced clearance seen in ESKD. Expert image-guided single-pass lumbar puncture (LP) measured an opening pressure of 31 cm H2O. Exhaustive CSF testing was negative, with a white cell count of zero and protein of 211 mg/L.

**Figure 4.**
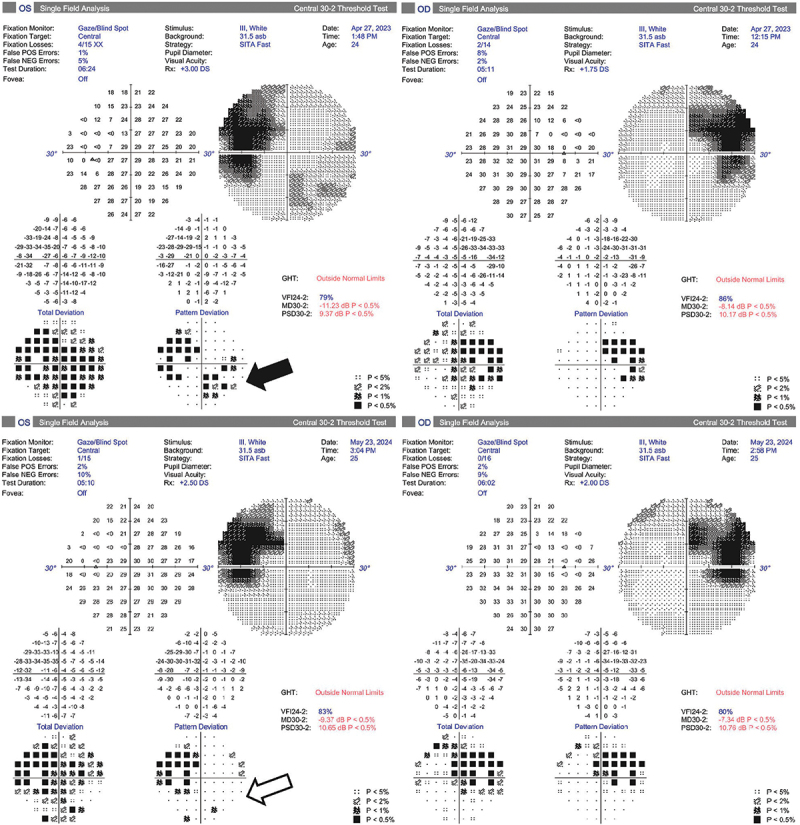
Evolution of the visual fields.

**Figure 5.**
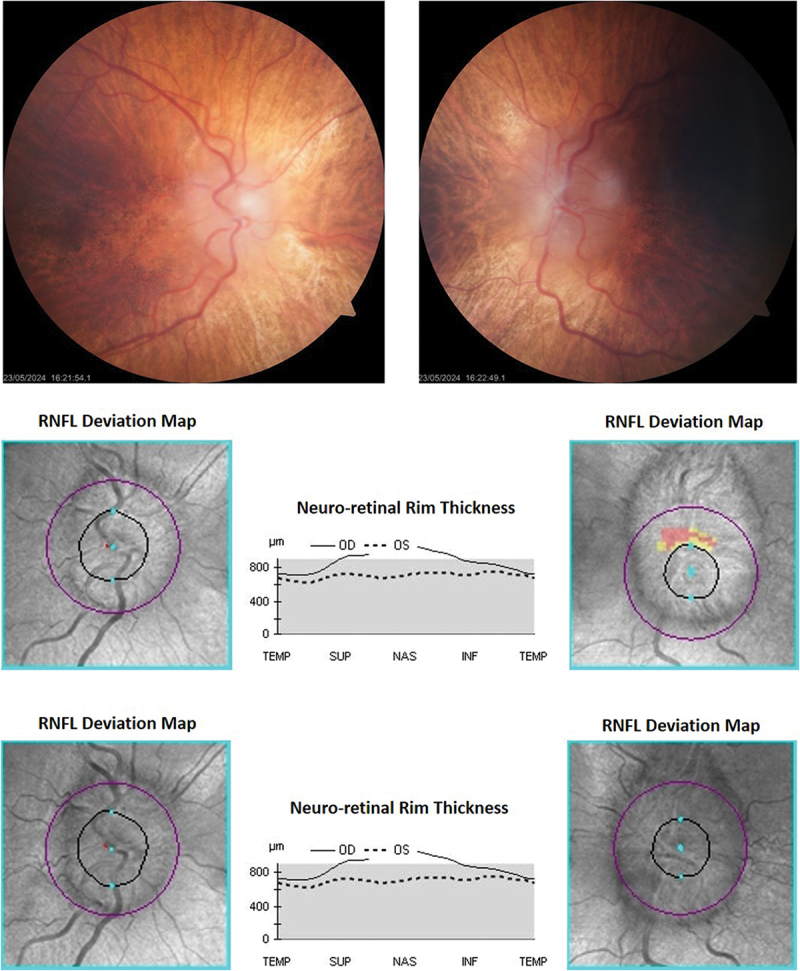
Optic discs after optic nerve sheath fenestration.

After LP, the patient noted the resolution of headache, and renally adjusted acetazolamide was commenced at 250 mg BD plus topiramate 25 mg BD. However, a new, reproducible left field loss developed in the form of an inferonasal defect (Figure 4), consistent with progressive optic disc pathology. Bilateral optic nerve sheath fenestration (ONSF) was performed, and the new field loss resolved (Figure 4). There was no change in the lateral extent of the disc swelling at follow-up 12 months after ONSF (Figure 5), but the degree of disc elevation was less on fundoscopy, and the patient was able to discontinue acetazolamide and topiramate without deterioration. Actual RNFL measurements were unreliable throughout, due to the gross disc swelling. Central acuity remained stable throughout, as did the GCL.

Top row: New reproducible left field loss developed (solid arrow), despite LP and renally adjusted acetazolamide and topiramate.

Bottom row: The field loss reversed within weeks of bilateral optic nerve sheath fenestrations (outlined arrow). The fields remained at baseline 12 months later.

Top row: 12 months after bilateral optic nerve sheath fenestrations, the lateral extent of disc swelling was unchanged relative to presentation. However, the degree of elevation was less on fundoscopy.

Middle row: The enface OCT images of the discs at presentation. RNFL measurements were unreliable throughout, due to the gross disc swelling.

Bottom row: The enface OCT images of the discs 12 months after bilateral optic nerve sheath fenestrations. Apparent improvement in the lateral extent of the left disc swelling after surgery is an optical illusion, due to variation in the position of the OCT scanning circle and scan quality. However, the degree of elevation was less on fundoscopy.

## Discussion

Alagille syndrome (ALGS) is an autosomal dominant, multiple-malformation syndrome of variable penetrance.^1^ With a prevalence of 1:30,000 to 1:70,000, it is caused by mutations in the JAG1 and NOTCH2 genes. It can result in intrahepatic bile duct paucity as well as cardiac, renal, and vascular defects, “butterfly” vertebrae, and a broad forehead triangulating to a pointed chin. Usually diagnosed within the first year of life, it typically causes cholestatic liver disease (jaundice, hepatomegaly, and pruritus).^2^

**Figure 6.**
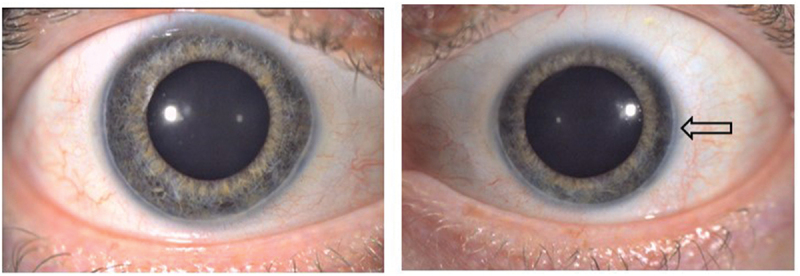
Our patient demonstrated bilateral posterior embyrotoxin (arrow), a classic finding in ALGS in which Schwalbe’s line is anteriorly displaced and thickened. The pupils are pharmacologically dilated here.

A range of ocular conditions are seen,^3^ most commonly posterior embryotoxon in some 70%, including our patient (Figure 6). Diffuse mottled fundus hypopigmentation is found, and mosaic iris stromal hypoplasia. Central acuity is usually well preserved, with only one or two lines’ reduction on average.^4^

Optic disc changes are very common, with 91% having disc elevation due either to drusen^5^ or glial proliferation,^6^ although true papilledema is reported in children.^7^ Further neuro-ophthalmic manifestations include cerebral arterial and venous abnormalities^8^ - unrecognized cerebral aneurysm may lead to sudden death.^9^

### Pseudopapilledema or true papilledema?

Diagnosing the cause of this patient’s swollen discs was laborious. Calcified drusen were excluded by B-scan and CT. Glial proliferation with possible uncalcified drusen was considered an incomplete diagnosis, given peripapillary hemorrhage at presentation when hemoglobin, platelets, and iron were normal. With headache, gross blind spot enlargement and transverse sinus abnormalities, it was deemed prudent to assess further for causes of papilledema, in particular idiopathic intracranial hypertension (IIH) – while only 2% of IIH occurs in men, it likely to be more severe than in women.^10^ Lumbar puncture was therefore considered necessary, both to measure the opening pressure, and to examine the CSF constituents.

Since LP confirmed elevated CSF pressure at 31 cm H2O, the patient did have true papilledema, as well as pseudopapilledema due to glial proliferation and perhaps drusen. Normal CSF constituents pointed away from inflammatory, infective, and demyelinating causes of disc swelling. The patient had adequate iron levels and was not on any relevant medication, so we speculated that the locus of defective CSF drainage may be the arachnoid villi or glymphatics,^11^ possibly as part of ALGS. While procedural intervention reversed the emerging field loss, it did not lead to a decline in the disc swelling, which was thought due to the structural abnormalities of glial hypertrophy and perhaps drusen, plus the presumed chronicity of papilledema. It was unclear if the patient’s reduced but stable GCL reflected macular atrophy or chronic papilledema.

### Further complexities

In this warfarinised patient, it was felt highly desirable to avoid CSF shunting to treat his elevated CSF pressure, given the high revision rate that shunting carries^12^ - 19% of shunt patients can expect a revision in the first 6 months, and 85% over their lifetime. Hence optic nerve sheath fenestration was deployed. Transverse sinus stenting was not a relevant consideration, as there was no clinically significant stenosis gradient.

The patient’s co-morbidities demonstrably complicated his neuro-ophthalmic care. The withdrawal of warfarin for LP and later ONSF created a potentially life-threatening risk of thromboembolism. Management of the patient overall was a major logistic exercise, requiring prolonged hospitalization and extensive collaboration across multiple specialties.

## Conclusion

Pseudopapilledema and papilledema can co-exist in Alagille syndrome. Adequately distinguishing them ensures that the emerging visual threat from papilledema is not overlooked. Treatment of the papilledema will be significantly influenced by ALGS co-morbidities, and patients should be screened for life-threatening cerebral aneurysms.
